# A 6.49-Mb inversion associated with the purple embryo spot trait in potato

**DOI:** 10.1007/s42994-025-00197-5

**Published:** 2025-01-18

**Authors:** Pei Wang, Lin Cheng, Jun Pan, Lianlian Ma, Xiaojing Hu, Zhong Zhang, Dawei Li, Yanhui Zhu, Shiwei Chang, Pingping Yuan, Philip Kear, Ludivine Lassois, Guangtao Zhu, Sanwen Huang, Hui Du, Chunzhi Zhang

**Affiliations:** 1 Shenzhen Branch, Guangdong Laboratory of Lingnan Modern Agriculture, Key Laboratory of Synthetic Biology, Ministry of Agriculture and Rural Affairs, Agricultural Genomics Institute at Shenzhen, Chinese Academy of Agricultural Sciences, Shenzhen, 518120 China; 2 Yunnan Key Laboratory of Potato Biology, The AGISCAAS-YNNU Joint Academy of Potato Sciences, Yunnan Normal University, Kunming, 650500 China; 3 National Key Laboratory of Tropical Crop Breeding, Chinese Academy of Tropical Agricultural Sciences, Haikou, 571101 China; 4 Plant Genetics and Rhizosphere Processes Laboratory, TERRA Teaching and Research Center, University of Liège, Gembloux Agro-Bio Tech, Gembloux, 5030 Belgium; 5 National Key Laboratory of Crop Genetic Improvement, Huazhong Agricultural University, Wuhan, 430070 China; 6 International Potato Center (CIP) China Center for Asia Pacific (CCCAP), Beijing, 102199 China

**Keywords:** Potato, Embryo spot, Anthocyanin accumulation, Inversion

## Abstract

The embryo spot trait leads to a deep purple or reddish coloration at the base of the cotyledons of the embryo, visible on both sides of flat potato (*Solanum tuberosum*) seeds. This trait has long been used by potato researchers and breeders as a morphological marker during dihaploid induction. The formation of embryo spots reflects the accumulation of anthocyanins, but the genetic basis of this trait remains unclear. In this study, we mapped the embryo spot trait to a 6.78-Mb region at the end of chromosome 10 using an F_2_ population derived from a cross between spotted and spotless plants. The recombination rate in the candidate region is severely suppressed, posing challenges for the map-based cloning of the underlying gene and suggesting large-scale rearrangements in this region. A de novo genome assembly of the spotted individual and a comparative genomic analysis to the reference genome of spotless potato revealed a 6.49-Mb inversion present in the spotted plant genome. The left breakpoint of this inversion occurred in the promoter region of an R2R3 MYB transcription factor gene that is highly expressed in the cotyledon base of spotted embryos but is not expressed in that of spotless embryos. This study elucidated the genetic basis for embryo spot formation in potato and provides a foundation for future cloning of the causative gene.

**Supplementary Information:**

The online version contains supplementary material available at 10.1007/s42994-025-00197-5.

## Introduction

Most cultivated potatoes (*Solanum tuberosum* L.), a major tuber crop, are clonally propagated autotetraploid plants (2*n* = 4*x* = 48) with a highly heterozygous genome (Bao et al. 2022). The complexity of tetrasomic inheritance has limited the genetic dissection of desired breeding products; in addition, traditional propagation via underground tubers also faces great challenges, including its high cost for tuber seeding and susceptibility to diseases (Lindhout et al. 2018; Stokstad 2019). Re-inventing potato as an inbred line-based diploid crop and propagation via true seeds have been proposed as potential and feasible strategies (Lindhout et al. 2011; Li et al. 2013; Jansky et al. 2016; Stokstad 2019). A rapid and efficient means to generating homozygous diploid inbred lines is through doubled haploid (DH) technology based on in vivo maternal haploid induction (Chaikam et al. 2019). The efficient identification of haploid individuals is essential for accelerating DH technology.

In potato, several haploid inducers (HIs) can be used as pollen donors, such as ‘phu1.22’ (PI225682), ‘IvP35’, ‘IvP48’, ‘IvP101’, and ‘PL4’ (CIP596131.4) (Hutten et al. 1993; Peloquin et al. 1996; Van Breukelen et al. 1977; Ordoñez et al. 2021). All these potato HI lines are homozygous dominant for anthocyanin pigments that are visible as an embryo spot on the botanical seed and as nodal bands on the stem of young plants (Hermsen and Verdenius 1973). This visual marker allows the easy distinction of haploids/dihaploids from hybrids at the seed stage, with seeds lacking the embryo spots being desired. However, the genetic basis of the morphological marker is not clear.

Anthocyanin biosynthesis defines a complex pathway that includes structural genes encoding enzymes responsible for pigment production and regulatory genes encoding transcription factors (TFs) regulating the expression of structural genes. In tetraploid potato tubers, anthocyanin biosynthesis is controlled by three loci, namely, *D* (developer), *P* (purple), and *R* (red). The *P* and *R* loci are structural genes, encoding the enzymes flavonoid-3′,5′-hydroxylase (F3′5′H) (Jung et al. 2005) and dihydroflavonol 4-reductase (DFR) (De Jong et al. 2003; Zhang et al. 2009), while *D* is a regulatory gene, encoding the R2R3 MYB TF ANTHOCYANINS (StAN2) and regulating expression of multiple anthocyanin structural genes in tuber skin (Jung et al. 2009). Similarly, the *I* (inhibitor), *R*, and *P* loci have also been described as affecting anthocyanin accumulation in diploid potato (Dodds and Long 1955, 1956), with *I* from diploid potato being equivalent to locus *D* from tetraploid potato. *P* was shown to be epistatic to *R* (De Jong 1991). The formation of the potato embryo spot is due to the interaction of two loci: *P* controls the production of purple pigments, and *B* restricts the distribution of pigmentation to the seed spot. A series of alleles exist for the *B* locus, namely, *B*^*d*^ > *B*^*c*^ > *B*^*b*^ > *B*^*a*^ > *b* (in order of dominance), for the control of pigment distribution to the floral abscission layer, tuber eyebrow, embryo spot, and nodal bands (Dodds and Long 1956). The gene responsible for the embryo spot may therefore have pleiotropic actions, as the plants produced from spotted seeds also present purple rings or bands at the bottom of their leaves and leaflets, scale leaves of stolons, tuber eyebrows, and floral abscission layers (Hermsen and Verdenius 1973). The *B* locus was recently mapped to chromosome 10 (chr10) by a k-mer-based bulked-segregant analysis approach (Sonsungsan et al. 2024), but the underlying gene is still unknown.

In this study, we performed a bulked-segregant analysis and detected two loci located on chr10 and chr11, respectively, controlling the formation of the embryo spot using an F_2_ population between a spotted potato line and a spotless potato line. The signal on chr11 overlapped with the reported location of *P* (Jung et al. 2005). We thus focused on the chr10 interval and narrowed down the candidate locus to a 6.78-Mb interval, but further mapping was hindered by severe recombination suppression over this region. Genomic analysis revealed a 6.49-Mb inversion in the candidate region. This inversion was associated with the expression of an R2R3 MYB TF gene, S20-1H2_C010G365180. This study enhances our understanding of the genetic basis of embryo spot formation in potato.

## Results

### The embryo spot trait is controlled by two genetic loci

**Fig. 1:**
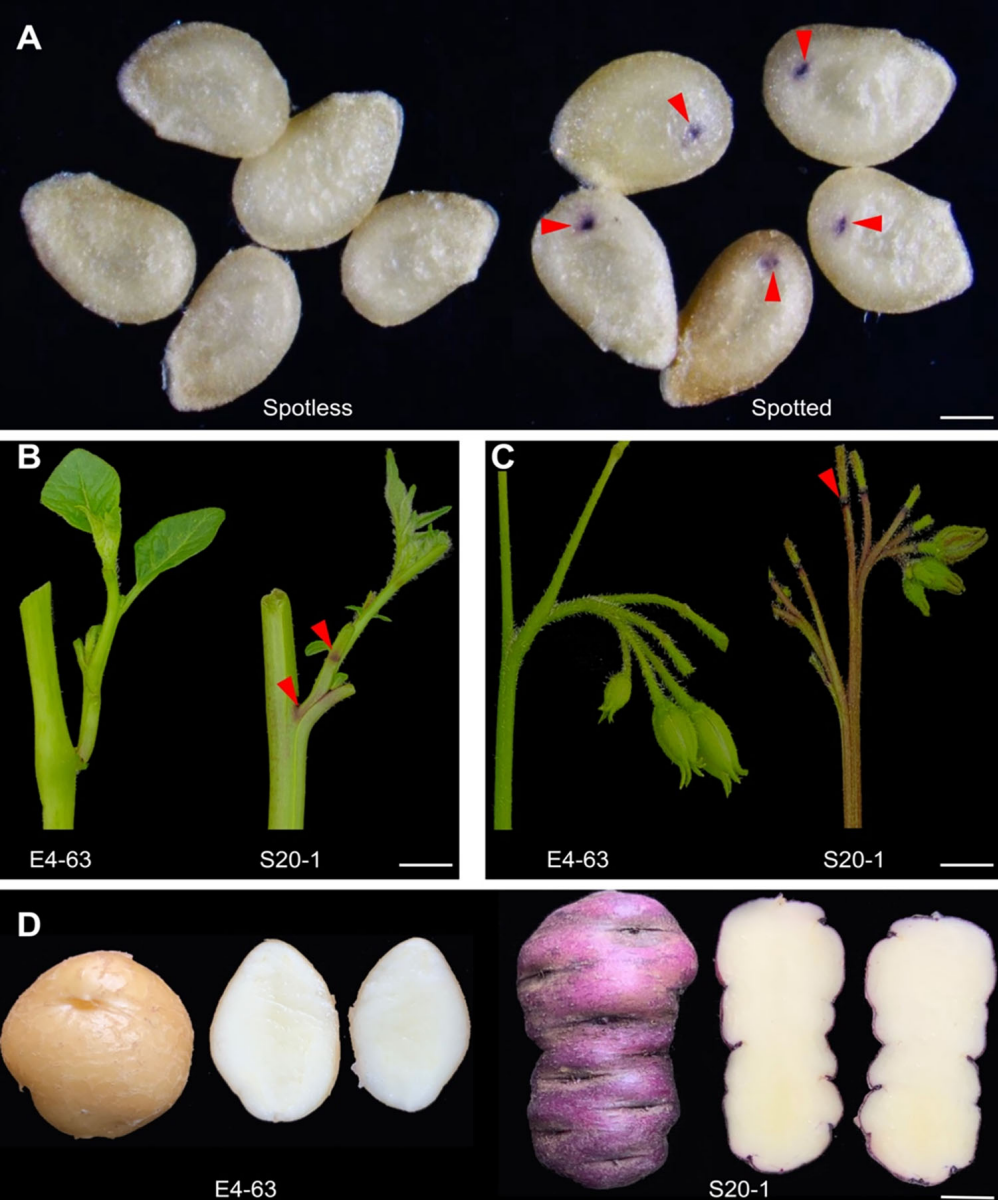
Representative photographs of spotted and spotless plants. **A** Spotless and spotted seeds. The red arrowheads indicate the deep purple pigmentation at the base of embryo cotyledons. Scale bar, 1 mm.** B** Pigmentation of the base of the petiole. The base of the petiole of E4-63 are green, while those of S20-1 are purple with extended green pigments on the surface and intense dark purple bands at the base of the petiole. Scale bar, 1 cm. **C** Pigmentation of the inflorescence. The inflorescence of E4-63 has green floral abscission layers, while that of S20-1 has purple floral abscission layers. Scale bar, 1 cm. **D** Pigmentation of tubers. The tuber skin of E4-63 is white, while that of S20-1 is purple. The tuber flesh color of both genotypes is white. Scale bar, 1 cm

The diploid clone S20-1 carries a morphological marker that results in embryos with a spot on hybrid seeds when used as the male parent in a cross. The embryo spot presents as a deep purple coloration at the base of cotyledons, which can be visible through the seed coat on both sides of the flat seeds (Fig. 1A). S20-1 also accumulates purple pigments at the base of the petiole (Fig. 1B), the floral abscission layers (Fig. 1C), and tuber skin (Fig. 1D). To investigate the genetics architecture of the embryo spot trait, we crossed the diploid inbred line E4-63 (as a female parent) to S20-1(as a male parent). The F_1_ progeny from this cross showed a 1:1 segregation ratio for the embryo spot phenotype, indicating that the locus controlling this trait is likely to be in a heterozygous state in S20-1, as E4-63 is highly homozygous and does not present the embryo spot trait itself (Zhang et al. 2021). We allowed the F_1_ clone I-8 showing the embryo spot phenotype to self-pollinate to generate a segregating F_2_ population. The segregation ratio of spotted and spotless seeds in this F_2_ population approached 9:7 (995:736, *χ*^2^ = 1.02, *P* = 0.31), suggesting that the embryo spot trait in this population is controlled by two independent loci.

**Fig. 2:**
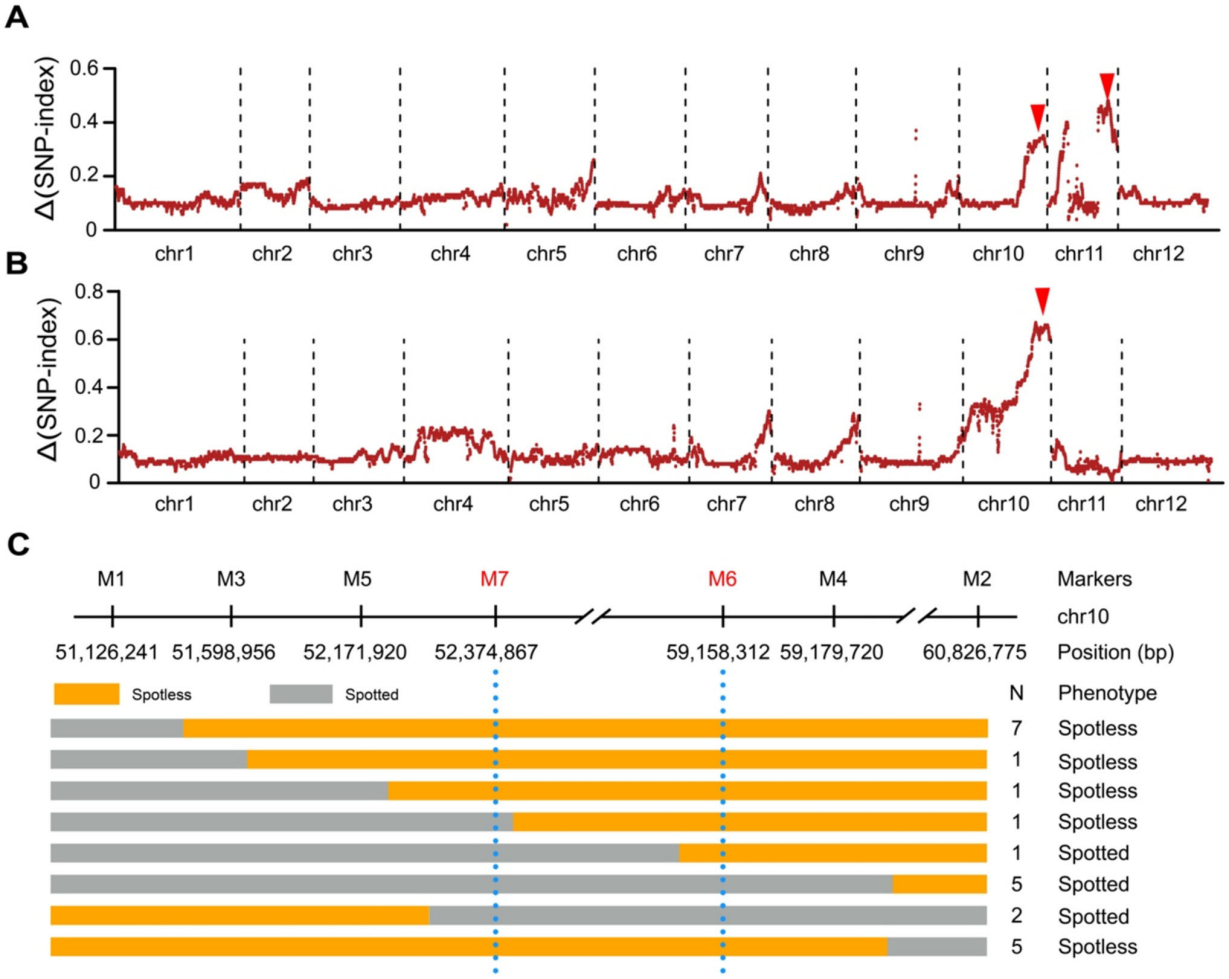
Genetic mapping of genes controlling embryo spot formation in potato seeds. **A** Bulked-segregant analysis sequencing (BSA-seq) analysis identified two candidate loci on chromosomes 10 (chr10) and 11. **B** BSA-seq analysis identifying the *B* locus on chr10 by selecting F_2_ individuals homozygous for the dominant *P* genotype. The red arrowheads in (**A**) and (**B**) indicate the peaks that correspond to the genomic loci controlling the embryo spot trait. **C** Fine mapping of the *B* locus. N indicates the number of recombinants with the indicated genotypes

To map the genes controlling the embryo spot trait, we collected tissue from 20 seedlings with spotted seeds and nodal anthocyanin bands in young plants, as well as 20 seedlings without spots or anthocyanin bands and pooled the genomic DNA of these seedlings to assemble a spotted pool and a spotless pool for bulked-segregant analysis sequencing (BSA-seq). We generated 37 Gb of sequencing reads for the spotted pool and 36 Gb for the spotless pool. We aligned the clean reads to the reference potato genome (DM6.01) to identify single-nucleotide polymorphisms (SNPs). By subtracting the SNP-index of the spotless pool from that of the spotted pool, we detected two Δ(SNP-index) signals, one on chr10 (chr10: 50.90–58.00 Mb) and one on chr11 (chr11: 36.41–42.01 Mb) (Fig. 2A). The locus on chr10 overlaps with the location of the previously reported *D*/*I* genes, which regulates the expression of anthocyanin biosynthesis genes (Jung et al. 2009). Similarly, the locus on chr11 contained *P*, encoding F3′5′H and controlling the production of purple pigments in potato tissues (Jung et al. 2005). As the formation of the embryo spot is due to the accumulation of purple pigments at the base of the cotyledons in the seed, we speculated that the transcriptional regulation of anthocyanin structural genes located on chr10 might be a key factor behind the embryo spot trait. To focus our search of this hypothetical TF gene, we chose another set of 20 spotted individuals and 20 spotless individuals that were all homozygous for the dominant genotype at the *P* locus from the F_2_ population to construct new pools fixed for *P* on chr10 for another BSA-seq analysis. The two new pools generated 36 Gb of sequencing reads, the analysis of which showed only one signal on chr10 (Fig. 2B). Previous studies have shown that the *B* locus, essential for anthocyanin pigment accumulation in potato seeds, is closely linked to the *I* locus on chr10 (Dodds and Long 1956; Van Eck et al. 1994). We therefore also designated this locus on chr10 as the *B* locus.

### Severe recombination suppression hampers the positional cloning of *B*

To clone the *B* gene, we developed high-density molecular markers in the candidate region on chr10 based on heterozygous insertion/deletion (InDel) polymorphisms identified in the genome of the F_1_ clone I-8. First, we used 1,731 F_2_ individuals (spotted: 995 and spotless: 736) to narrow down the *B* interval. To eliminate the influence of *P*, we designed InDel markers (Supplementary Table 1) to select individuals with the *P*^−^genotype. We thus obtained 1,056 F_2_ individuals with the *P*^−^ genotype for detection of recombination events across the mapping interval, yielding 23 recombinants between the flanking markers M1 and M2 (Fig. 2C). Among these 23 recombinants, eight individuals showed a purple embryo spot on the true seeds and a nodal anthocyanin band in young plants, while the other 15 plants did not have a purple embryo spot or an anthocyanin band. We developed additional markers (Supplementary Table 1) to genotype these individuals with recombination events and narrowed down the location of *B* to a 6.78-Mb interval (chr10: 52.37–59.15 Mb) between markers M6 and M7 (Fig. 2C). However, we failed to identify more recombinants between M6 and M7 when genotyping an additional 1,000 F_2_ clones. In our previous study, we analyzed five diploid F_2_ populations to assess the genome-wide meiotic crossover landscape in potato, revealing a high recombination rate at the end of chr10 (Jiang et al. 2023). Thus, we concluded that the F_2_ population used in this study exhibits severe recombination suppression in the candidate region, hampering the positional cloning of *B*.

### Detection of a 6.49-Mb inversion at the *B* locus in the S20-1 genome

To identify structural variations (SVs) that may affect the recombination rate, we examined a de novo genome assembly of the female parent E4-63 (Zhang et al. 2021); however, we did not identify any large SVs in the candidate region relative to the DM6.01 reference genome. We thus speculated that the recombination suppression might be caused by SVs present only in the male parent S20-1. Therefore, we performed a de novo assembly of the S20-1 genome.

The estimated genome size of S20-1 is ~ 744 Mb with a heterozygosity rate of 0.96%. Our initial contig-level assembly using ~ 23.80 Gb (33 ×) of circular consensus sequencing (CCS) reads resulted in two haplotype assemblies of 1.55 Gb in total (Supplementary Table 2). The total lengths of the initial haplotype 1 (Hap1) and haplotype 2 (Hap2) assemblies were 791.30 Mb with an N50 contig length of 18 Mb for Hap1 and 762.30 Mb with an N50 contig length of 18.60 Mb for Hap2. We then scaffolded the initial assembly and ordered with RagTag, using a chromosome-level assembly of the close relative, DM6.01, as a reference genome to reconstruct pseudochromosomes for each haplotype. According to gene prediction and functional annotation analyses, we annotated 42,736 genes for Hap1 and 42,928 genes for Hap2 (Supplementary Table 3). We estimated the completeness of these two assemblies by calculating their Benchmarking Universal Single-Copy Orthologs (BUSCOs) scores, achieving 98.90% and 99% for Hap1 and Hap2, respectively (Supplementary Table 4), indicating the high contiguity and completeness of the S20-1 genome assemblies.

**Fig. 3:**
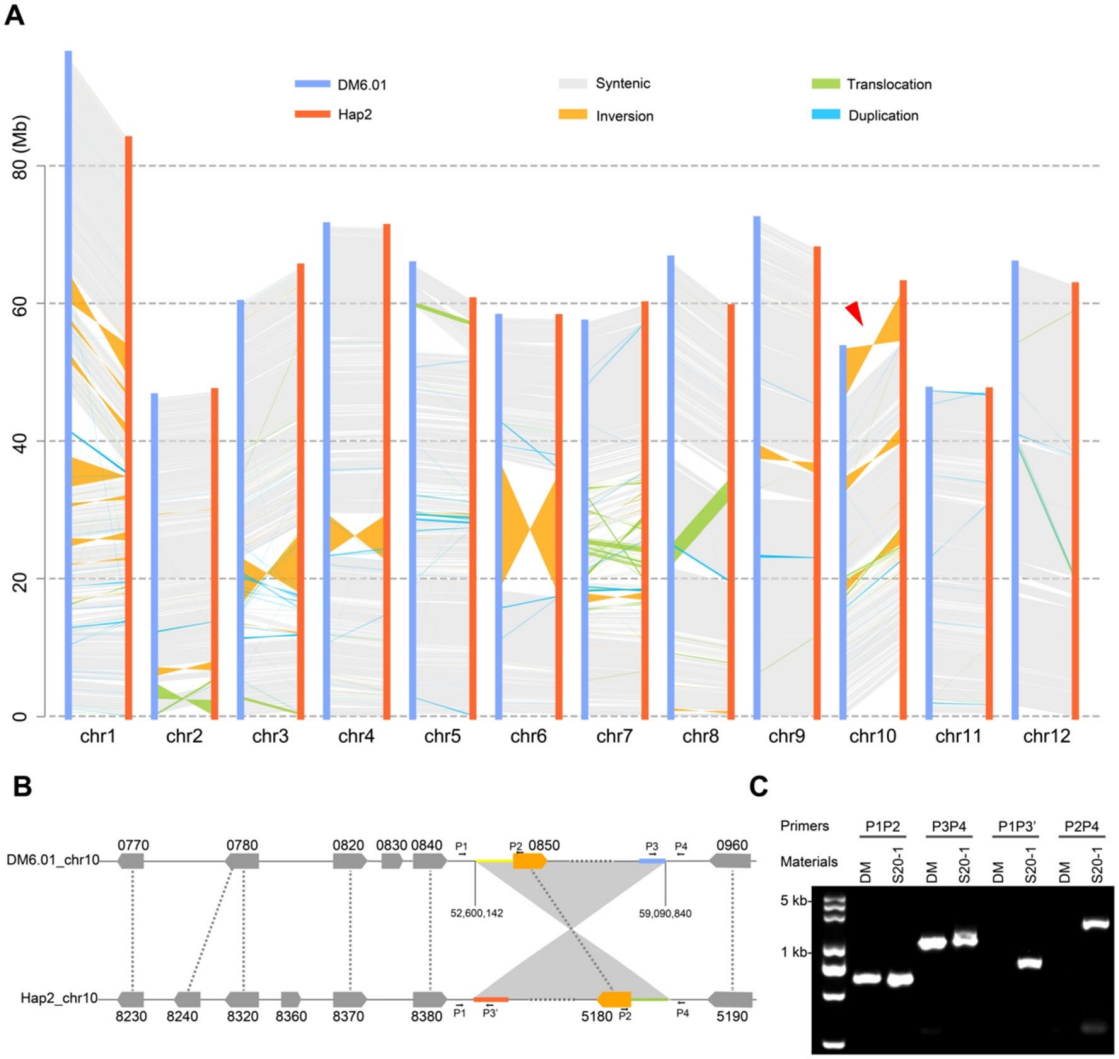
De novo genome assembly of the S20-1 genome reveals a large inversion at the end of chr10. **A** Identification of genomic variation between haplotype 2 (Hap2) of the S20-1 genome compared to the DM6.01 reference genome. An inversion at the end of chr10 is indicated by the red arrowhead. **B** Diagram of the inversion at the end of chr10 from Hap2 of S20-1. The blue, red, yellow, and green horizontal lines on each end of the inversion intervals indicate unaligned regions. **C** PCR validation of the inversion at the end of chr10 in Hap2 of S20-1. PCR products were analyzed by agarose gel electrophoresis

To accurately evaluate the divergence between the two haplotypes, we identified polymorphisms between the 12 pairs of homologous chromosomes, leading to the identification of 2,381,332 SNPs, 529,689 InDels (1 ~ 50 bp), and 16,913 SVs (insertion, translocation, duplication, inversion, and deletion, > 50 bp). There were 106 inversions between the two haplotypes, accounting for approximately 64 Mb of genome sequence (Supplementary Table 5). Notably, we noticed a 6.49-Mb inversion at the end of chr10 specific to Hap2 (Fig. 3A), which is likely the cause of the observed recombination suppression at the *B* locus.

To verify whether the detected 6.49-Mb inversion was a true rearrangement or an assembly error, we designed four pairs of primers around the inversion breakpoints based on the contig-level assembly and conducted PCR tests (Fig. 3B, C). Primer sets P1P2 and P3P4 successfully produced an amplicon of the expected size when using genomic DNA from DM and S20-1 as template; however, the primer sets P1P3’ and P2P4 failed to produce an amplicon when using genomic DM DNA as template (Fig. 3C). By contrast, the same primer sets P1P3’ and P2P4 successfully amplified an amplicon from S20-1 genomic DNA (Fig. 3C), confirming the presence of the large inversion in Hap2 of S20-1 (Fig. 3A).

### The 6.49-Mb inversion affects the expression of an R2R3 MYB transcription factor gene

**Fig. 4:**
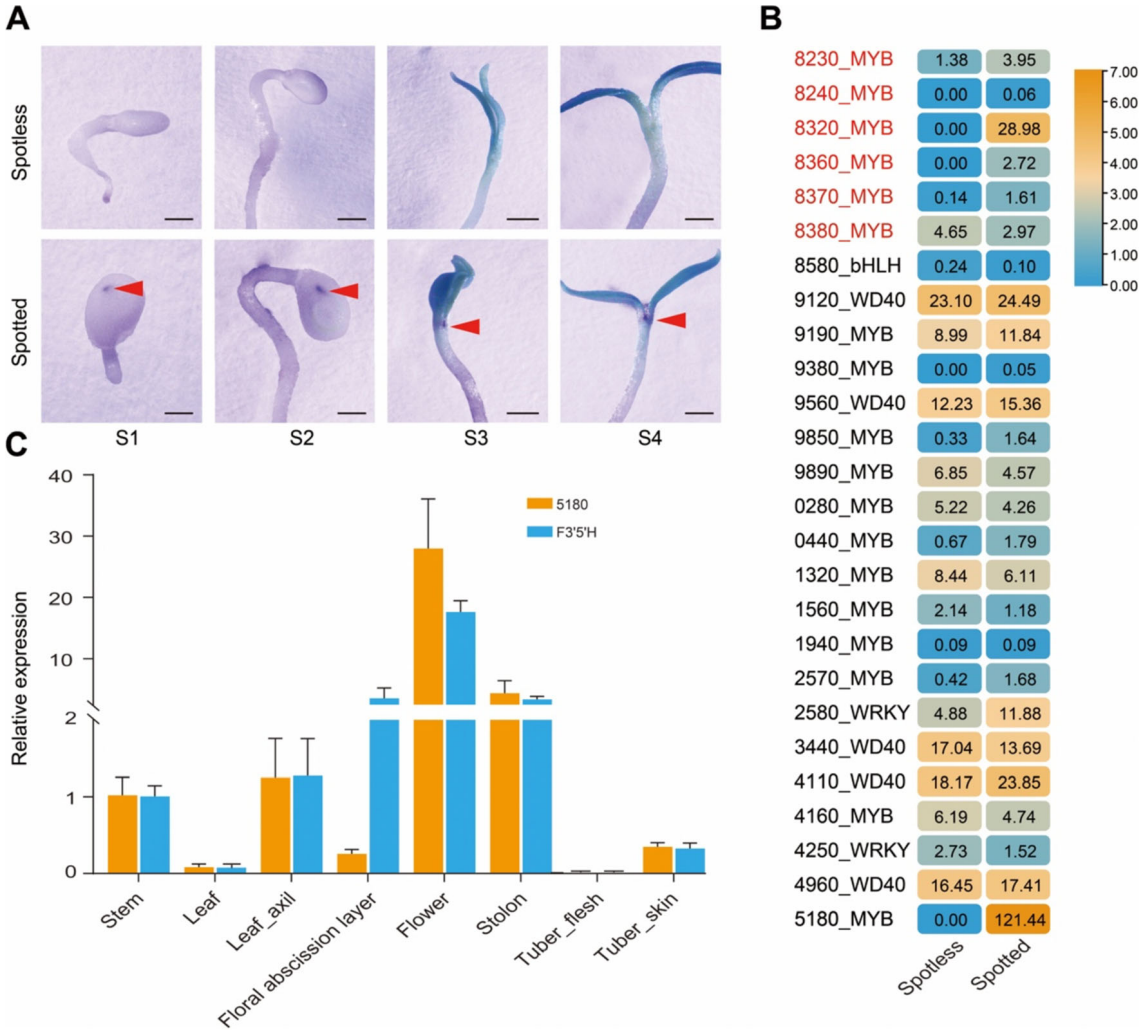
Analysis of candidate genes for embryo spot formation. **A** Representative photographs of spotless and spotted seeds at different germination stages: S1–S4 (stage 1–stage 4). The red arrowheads point to the purple zone in the cotyledon node that is visible as the purple embryo spot in seeds. Scale bars, 1 mm. **B** Heatmap representation of the expression levels for MYB transcription factor (TF) genes, bHLH TF genes, WD40 genes, and WRKY TF genes located between markers M6 and M7. The genes names in red indicate genes located outside the inversion, while others are located within the inversion region. The numbers in the boxes are the FPKM (Fragments Per Kilobase of transcript per Million mapped reads) values. **C** Expression analysis of S20-1H2_C010G365180 (5180) and *F3′5'H* in different tissues of S20-1 as determined by RT-qPCR. Data are means ± SD from three biological replicates

Anthocyanin biosynthesis is typically regulated by complex interactions among MYB TFs, basic helix–loop–helix (bHLH) TFs, and WD40 proteins (Baudry et al. 2004; Patra et al. 2013). In addition, WRKY TFs also participate in regulating anthocyanin biosynthesis (Tao et al. 2024). To identify the possible candidate genes for the formation of the embryo spot, we performed transcriptome deep sequencing (RNA-seq) of total RNA extracted from the cotyledon node zone of seedlings germinated from spotted or spotless seeds (Fig. 4A). Within the 6.78-Mb interval between markers M6 and M7, there are 18 MYB TF genes, 1 bHLH TF gene, 5 WD40 genes, and 2 WRKY TF genes, of which 20 are located within the inversion region (Fig. 4B). Transcriptome analysis revealed that two MYB TF genes, S20-1H2_C010G358320 (8320) and S20-1H2_C010G355180 (5180), are highly expressed in spotted seedlings but were not expressed in spotless seedlings. Notably, one breakpoint of the 6.49-Mb inversion occurred in the promoter region of the 5180 gene (Fig. 3B). *Stan2*, an allele of 5180, was previously identified as the candidate gene of the *D*/*I* locus (Supplementary Fig. S1), which regulates anthocyanin biosynthesis in tuber skin (Jung et al. 2009). Reverse-transcription quantitative PCR (RT-qPCR) analysis showed that 5180 and *F3′5'H* (the *P* gene) are mainly co-expressed in multiple tissues where anthocyanins accumulate, such as the stem, leaf axil, floral abscission layer, flowers, stolons, and tuber skin (Fig. 4C). Therefore, we speculate that S20-1H2_C010G365180 might be the candidate gene for the *B* locus that contributes to the formation of the embryo spot.

## Discussion

The haploid induction system has been successfully utilized in potato germplasm innovation and breeding. All reported haploid inducer lines, such as ‘IvP35’, ‘IvP101’, and ‘PL4’, have a common morphological marker, the embryo spot in the seed, thereby facilitating the identification of chromosome-reduced progeny. In this study, we identified two genetic loci controlling the formation of embryo spot: one locus on chr11 that contains the *P* gene (Jung et al. 2005), and the other locus on chr10, overlapping with the *B* locus. These findings are consistent with the previous reports (Dodds and Long 1956; Sonsungsan et al. 2024). The *B* locus is linked to the *D*/*I* locus, the *Pigmented tuber flesh* (*Pf*) locus, and the *F* locus, which are required for the tissue-specific accumulation of anthocyanins in tuber skin, tuber flesh, and flowers, respectively (Dodds and Long 1956; Hermsen and Verdenius 1973; De Jong 1991). Among them, *D* encodes the R2R3 MYB TF Stan2 (Jung et al. 2009) and the candidate gene for the *F* locus is also an R2R3 MYB TF gene called *StFlAN2,* corresponding to gene ID Soltu.DM.10G020780 (Laimbeer et al. 2020). In this study, we identified S20-1H2_C010G365180*,* which also encodes an R2R3 MYB TF and is an allele of *D/Stan2*, might be the candidate gene of the *B* locus. The genomic region of the *B*, *I*, *Pf*, and *F* loci contains multiple MYB TF genes, which are key regulators of anthocyanin biosynthesis (Liu et al. 2015). Thus, we hypothesize that the different alleles of these MYB TF genes may be involved in regulating the accumulation of anthocyanins in various potato tissues.

Large chromosomal inversions (INVs), an important type of structural variation, suppress recombination by hampering chromosome pairing (Wellenreuther and Bernatchez 2018). Inversions occur frequently in plants, contributing to the genetic basis of local adaptation and ecotypic differentiation (Li et al. 2022; Jin et al. 2023; Wang et al. 2023). In tomato (*Solanum lycopersicum*), an inversion on chr10 disrupts the *LUTESCENT2* (*L2*) gene, which affects tomato fruit color and ripening speed (Li et al. 2021). Based on the rice (*Oryza sativa*) pan-genome inversion map, multiple differentially expressed genes were identified that are located either within the inversion or in the flanking regions of the inversion (Zhou et al. 2023). For example, one breakpoint of *OsINV10* is located in the promoter region of the MADS TF gene *MADS56*, strongly affecting the expression level of this gene, which may be related to the heat tolerance of rice (He et al. 2023). Here, we discovered a 6.49-Mb inversion at the *B* locus, which suppressed recombination and hampered the map-based cloning of the causal gene. This inversion altered the promoter of S20-1H2_C010G355180. This gene was expressed in all pigmented tissues of spotted plants, including stems, the leaf axil, the floral abscission layer, flowers, stolons, and tuber skin, but was undetectable in spotless plants. The *P* gene shows a similar expression pattern, indicating that these two genes may collectively control the anthocyanin accumulation. The genotype at the *P* locus is therefore likely to be essential to further functional analysis of potential candidate genes of *B* by genetic transformation of other potatoes. Importantly, these findings elucidated the inheritance patterns of the embryo spot in potato and predicted a candidate gene for the *B* locus. In the future, after candidate genes are validated, we can combine both causal genes for the *B* and *P* loci to develop a set of morphological markers for potato embryo spots, which can be utilized in the potato haploid induction system to accelerate the creation of potato inbred lines.

## Materials and methods

### Plant materials

The female parent E4-63 is a highly homozygous diploid inbred line containing the *S-locus inhibitor* gene developed in our previous study (Zhang et al. 2021). The male parent S20-1 accumulates purple pigments in axils, the floral abscission layer, and tuber skin. This clone can produce spotted hybrid seeds when used as a male parent. Self-pollination of an F_1_ clone I-8 (spotted), derived from a cross between E4-63 and S20-1, generated an F_2_ mapping population, which was used for phenotyping and mapping the loci controlling the formation of the pigmented embryo spot.

### Bulked-segregant analysis of the embryo spot trait

Genomic DNA was extracted from the fresh leaves of F_2_ plants using the CTAB method (Murray and Thompson 1980). Two pools, each containing 20 spotted plants with spotted seeds and nodal anthocyanin bands in young plants and 20 spotless plants without spots or anthocyanin bands, were used for sequencing. The Watchmaker DNA Library Prep Kit with Fragmentation (CAS: 7K0019-096) was used to prepare the library. Sequencing was performed on the Illumina X Ten platform, and the double-ended sequencing program (PE) was run to obtain 150-bp double-ended sequencing reads. Fastp (v0.21.0) (Chen et al. 2018) was used to filter out low-quality data, including reads with ≥ 10% unidentified nucleotides (Ns) and reads with > 50% of bases having a Phred quality < 5, as well as to remove adapter sequences from the raw reads. Short reads were aligned to the reference potato genome (DM6.01) (Pham et al. 2020) using the Burrows–Wheeler Aligner (Li and Durbin 2009). SNPs were identified using SAMtools and BCFtools software (Li 2011), with the filtering criteria of sequencing depth being between one-third and three times the average sequencing depth and a mapping quality of at least 30. Taking the haplotype of E4-63 as the reference, SNP-index values were calculated using a sliding window method (window size, 1 Mb; step, 10 kb). The Δ(SNP-index) was calculated to identify the candidate loci (Takagi et al. 2013).

### Development of PCR-based markers and mapping of the embryo spot trait

Fruits were harvested 60 days after self-pollination the F_1_ clone I-8, and well-developed seeds were phenotyped for the presence of the embryo spot. The seeds were then treated with 2 mg/mL gibberellic acid (GA3, Sigma) for 1 day to break dormancy and were incubated on a damp filter paper at 20 °C for 7 days. After emergence, seedlings were transplanted into MS medium (Murashige & Skoog Basal Medium with Vitamins 2.17 g, sucrose 15 g, and Gelzan™ CM 3.12 g in 1 L H_2_O), and the presence of nodal anthocyanin bands was recorded. Only seedlings with the *P*^−^genotype were used for determination of recombination events. Primers to amplify InDel markers used for selecting the *P*^−^ genotype and for mapping the *B* locus were designed as previously described (Yang et al. 2020), and the PCR products were separated on an 8% (w/v) polyacrylamide gel.

### De novo genome assembly and annotation of the S20-1 genome

The circular consensus sequencing (CCS) reads for S20-1 were assembled with Hifiasm (Cheng et al. 2021) using default parameters. The initial output file from Hifiasm (v0.16) yielded two assemblies, a primary assembly for Hap1 and one for Hap2. The two primary assemblies were subsequently scaffolded and ordered with RagTag (Alonge et al. 2022) using a chromosome-level assembly from the closely related clone DM6.01 (Pham et al. 2020) to reconstruct pseudochromosomes for each haplotype. To assess the completeness of the assembly, a Benchmarking Universal Single-Copy Orthologs (BUSCO) (Simão et al. 2015) analysis was conducted. The annotation of gene structure and function was carried out as previously described (Tang et al. 2022).

### Identification of sequence variation between the two S20-1 haplotypes

The genome of the Hap2 assembly of S20-1 was first aligned to the Hap1 assembly of S20-1 with Minimap2 v2.17 (Li 2021) with default parameters to produce an SAM file. SyRI v1.5 (Goel et al. 2019) was employed to call variants from the above alignment to generate a variation calling format (VCF) file.

### PCR verification of the inversion from the two S20-1 haplotypes

The primers used for verifying the inversion were designed by Primer3 (https://github.com/primer3-org/primer3). PCR was conducted on an ABI Applied Biosystems with PrimeSTAR® GXL DNA Polymerase (Takara, Japan) under the following conditions: 95 °C for 5 min, followed by 35 cycles of 98 °C for 10 s, 58 °C for 15 s, and 68 °C for 2.5 min. The PCR products were separated on a 1.5% (w/v) agarose gel. The primers used are listed in Supplementary Table 1.

### Transcriptome analysis and reverse-transcription quantitative PCR (RT-qPCR)

The cotyledon node zone was collected from 1-week-old seedlings germinated from spotted or spotless seeds; each sample contained 50 plants. At the flowering stage, samples were collected from the stem, leaf, leaf axil, flower, and floral abscission layer of S20-1. At the mature stage, samples were collected from stolon, tuber skin, and tuber flesh of S20-1. Total RNA was extracted using an RNAprep Pure Plant Kit (Tiagen, China) following the manufacturer’s instructions.

The library was constructed by VAHTS Universal V6 RNA-Seq Library Prepkit for Illumina NR 604-01/02 (Vazyme, Nanjing, China). RNA sequencing was conducted by Annoroad Genomics (Beijing, China) using the MGI sequencing platform, generating paired-end 150-bp reads and producing 4 Gb of raw data. Fastp (v0.21.0) was used to remove the low-quality data (reads with ≥ 10% unidentified nucleotides (Ns) and reads with > 50% of bases having a Phred quality < 5) and adapter sequences from raw reads (Chen et al. 2018). The clean reads were mapped to the S20-1 genome using HISAT2 (Kim et al. 2015). The FPKM (Fragments Per Kilobase of transcript per Million mapped reads) values of each gene were calculated with StringTie (Pertea et al. 2015). The heat map of MYB TF genes, bHLH TF genes, WD40 genes, and WRKY TF genes within the 6.78-Mb interval between markers M6 and M7 was generated using their FPKM values with TBtools (Chen et al. 2020).

For RT-qPCR analysis, first-strand cDNA was prepared using a PrimerScript RT reagent kit (Takara, Japan). qPCR was conducted on an ABI Step One-Plus System with TB Green™ Premix Ex Taq™ GC (Takara, Japan) under the following conditions: 95 °C for 2 min, followed by 40 cycles of 95 °C for 5 s, 58 °C for 15 s, and 72 °C for 30 s. The *ACTIN* gene (Soltu.DM11G008990) was used as an internal reference gene. Three biological replicates were analyzed. The relative expression levels were calculated by the 2^−ΔΔCt^ method (Livak and Schmittgen 2001). The primers used for RT-qPCR are listed in Supplementary Table 1.

### Accessions numbers

Sequence data in this article can be found in the website (https://spuddb.uga.edu/dm_v6_1_download.shtml) under the following accessions number: *F3′5'H* (Soltu.DM.11G020990), 0770 (Soltu.DM.10G020770), 0780 (Soltu.DM.10G020780), 0820 (Soltu.DM.10G020820), 0830 (Soltu.DM.10G020830), 0840 (Soltu.DM.10G020840), 0850 (Soltu.DM.10G020850), and 0960 (Soltu.DM.10G020960). Additionally, data can be accessed at https://figshare.com/articles/dataset/Haplotype_resolved_genome_assembly_and_annotation_of_S20-1/26808739 under the following accession numbers: 8230 (S20-1H2_C010G358230), 8240 (S20-1H2_C010G358240), 8320 (S20-1H2_C010G358320), 8360 (S20-1H2_C010G358360), 8370 (S20-1H2_C010G358370), 8380 (S20-1H2_C010G358380), 8580 (S20-1H2_C010G358580), 9120 (S20-1H2_C010G359120), 9190 (S20-1H2_C010G359190), 9380 (S20-1H2_C010G359380), 9560 (S20-1H2_C010G359560), 9850 (S20-1H2_C010G359850), 9890 (S20-1H2_C010G359890), 0280 (S20-1H2_C010G360280), 0440 (S20-1H2_C010G360440), 1320 (S20-1H2_C010G361320), 1560 (S20-1H2_C010G361560), 1940 (S20-1H2_C010G361940), 2570 (S20-1H2_C010G362570), 2580 (S20-1H2_C010G362580), 3440 (S20-1H2_C010G363440), 4110 (S20-1H2_C010G364110), 4160 (S20-1H2_C010G364160), 4250 (S20-1H2_C010G364250), 4960 (S20-1H2_C010G364960), 5180 (S20-1H2_C010G365180), and 5190 (S20-1H2_C010G365190).

## Supplementary Information

Below is the link to the electronic supplementary material.

Supplementary file1 (DOCX 460 KB)

## Data Availability

The BSA-seq data are available at NCBI with BioProject accession number PRJNA1070059. The assembled S20-1 genome and annotation data can be found in the figshare platform (https://figshare.com/articles/dataset/Haplotype_resolved_genome_assembly_and_annotation_of_S20-1/26808739).
