# Supplementary material for: A 6.49-Mb inversion associated with the purple embryo spot trait in potato
Source: aBIOTECH. 2025 Jan 18;6(1):22–32. doi: 10.1007/s42994-025-00197-5 (PMC11889318; doi:10.1007/s42994-025-00197-5)
Supplement: Supplementary file 1 — Supplementary file1 (DOCX 460 KB) [file 42994_2025_197_MOESM1_ESM.docx]

**Supplementary Table 1 Primers used in this study**

| **Applications** | **Name** | **Forward sequence (5’-3’)** | **Reverse sequence (5’-3’)** |
| --- | --- | --- | --- |
| Fine mapping | M1 | ATCAAACTCAACGCTCTCAT | GATTTGGTGATCATTCTGCT |
|  | M2 | GTCATTGGAGTGTTAGGAGC | GCAACTTTTGGTCTTCTTCA |
|  | M3 | TCAGATGGAAAATTAGGCAC | GGTACAAACCGGATTACCTA |
|  | M4 | CGTTTTCGGCCATTAGATA | TTAGTGACGTGACAAAATGG |
|  | M5 | GCCAGAAGCTTAATTGAGAA | ATCTAAAACCCGATGCAGTA |
|  | M6 | ATGGGACTATATGTGTCAGC | ACTTACGAAGCTTGAACTCG |
|  | M7 | CCTAAAGAAGATGAGCATTG | TGTAGCTAAGAAAAGGGCAG |
| RT-qPCR | *ACTIN*  5180  *F3'5'H* | GGATCTTGCTGGTCGTGATTTAAC  CTCAGAAATTCACAAGCTCCATG  GATGAACATGAAGCAACTACC | CATAGGCAAGCTTTTCCTTCATG  CTTACATGAAGGTAGTGTTCTTT  AGATGAAGTGTCTGTACCAGC |
| Identifying the inversion | P1 | GATGTCGCATATTTGGAGCCTC  GTCACCTCTCTTGATATGTGGCC  AAGCTACTATGAATACAACTGCAC  GGTAGAAATAGACGTAAACCTTACCC  CTTGGGTGCTATCTTGATCAGGTC | |
|  | P2  P3  P3’  P4 |  |  |
| Identifying the *P* gene | chr11-p | TTATCCAATAAACGTGTCCCT | AGGAACTGTTGACTCTAATGC |

**Supplementary Table 2** **The summary of the Circular Consensus Sequencing (CCS) reads of S20-1**

| **CCS reads** | **S20-1** |
| --- | --- |
| Raw data (Gb) | 23.80 |
| Sequencing depth (×) | 33 |
| Average reads length (bp) | 16,935.60 |
| The number of reads | 1,406,267 |
| The summary of read lengths (bp) | 23,815,948,664 |

**Supplementary Table 3 The annotation statistics of S20-1**

| **ID** | **Gene count** | **Average gene length** | **Transcript counts** | **Transcript size** | **Mean exon size** | **Exon count per gene** | **Protein BUSCO (%)** |
| --- | --- | --- | --- | --- | --- | --- | --- |
| S20-1_H1 | 42,736 | 3,757 | 51,414 | 4,381 | 271 | 4.9 | 93.9% |
| S20-1_H2 | 42,928 | 3,721 | 49,667 | 4,220 | 270 | 4.8 | 93.9% |

**Supplementary Table 4 Haplotype-resolved chromosomal-level assemblies of S20-1**

|  | **Haplotype1** | **Haplotype2** |
| --- | --- | --- |
| Length of chromosomes (Mbs) | 791.30 | 762.30 |
| N50 | 18 | 18.60 |
| BUSCOs (%) | 98.90% | 99% |
| Complete BUSCOs (C) | 1,596 | 1,597 |
| Complete and single-copy BUSCOs (S) | 1,566 | 1,567 |
| Complete and duplicated BUSCOs (D) | 30 | 30 |
| Fragmented BUSCOs (F) | 3 | 3 |
| Missing BUSCOs (M) | 15 | 14 |
| Total BUSCO groups searched | 1,614 | 1,614 |

**Supplementary Table 5 The summary of structural variations in the S20-1 genome**

| **Variation_type** | **Count** | **Length_ref** | **Length_qry** |
| --- | --- | --- | --- |
| Inversions | 106 | 64,570,261 | 62,426,215 |
| Translocations | 1,804 | 29,547,349 | 30,025,009 |
| Duplications (reference) | 391 | 10,734,604 | - |
| Duplications (query) | 5,854 | - | 31,955,652 |
| Insertions | 407,395 (> 50 bp, 4,161) | - | 3,480,849 |
| Deletions | 131,052 (> 50 bp, 4,597) | 5,108,550 | - |

**
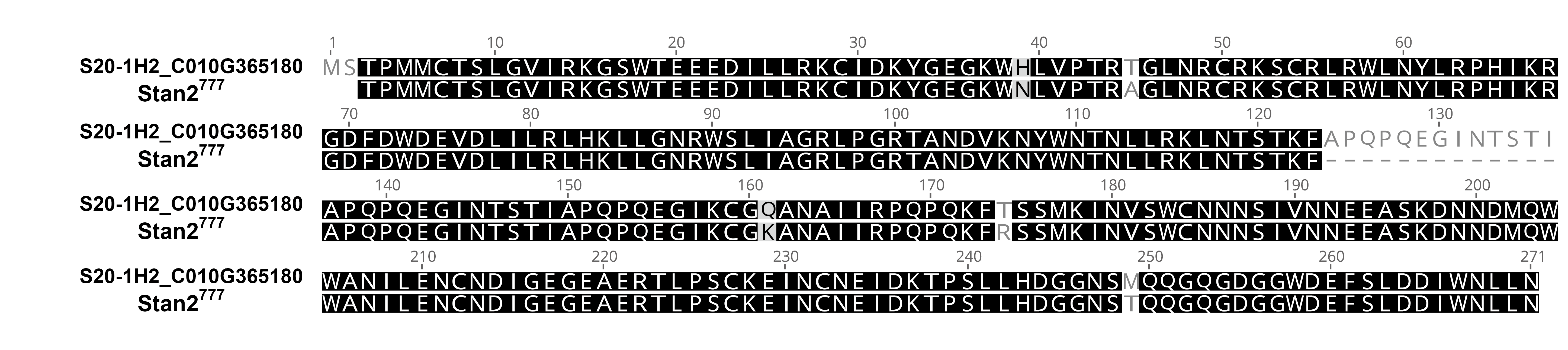
**

**Supplementary Fig. S1 Amino acid sequence alignment of S20-1H2_C010G365180 and Stan2**
